# Apo10 and TKTL1 in blood macrophages as non-invasive biomarkers for early detection of cervical cancer

**DOI:** 10.1007/s13402-025-01092-3

**Published:** 2025-07-28

**Authors:** Shuqing Wang, Zhiying Mao, Yuying Liu, Chunyan Lan, Lizhi Liu, Musheng Zeng, Chuanbo Xie

**Affiliations:** 1 Cancer Prevention Center, State Key Laboratory of Oncology in South China, Guangdong Provincial Clinical Research Center for Cancer, Sun Yat-sen University Cancer Center, Guangzhou, Guangdong 510060 China; 2 Department of Radiology, State Key Laboratory of Oncology in South China, Guangdong Provincial Clinical Research Center for Cancer, Sun Yat-sen University Cancer Center, Guangzhou, Guangdong 510060 China; 3 Department of Gynecologic Oncology, State Key Laboratory of Oncology in South China, Collaborative Innovation Center for Cancer Medicine, Sun Yat-sen University Cancer Center, Guangzhou, 510060 China; 4 Department of Experimental Research, State Key Laboratory of Oncology in South China, Guangdong Provincial Clinical Research Center for Cancer, Sun Yat-sen University Cancer Center, Guangzhou, Guangdong 510060 China; 5 Guangdong-Hong Kong Joint Laboratory for RNA Medicine, Guangzhou, Guangdong 510060 China

**Keywords:** Cervical cancer, Apo10, TKTL1, Screening

## Abstract

**Purpose:**

Apo10 and TKTL1 are tumor-associated markers reflecting impaired apoptosis and enhanced glycolysis respectively. This study aimed to evaluate the diagnostic potential of Apo10, TKTL1, and APT (a combination of Apo10 and TKTL1) in screening early-stage cervical cancer.

**Methods:**

A total of 152 patients with cervical cancer and 152 age-matched healthy controls were enrolled at Sun Yat-sen University Cancer Center from November 2020 to August 2023. Clinical data were collected from the Hospital Information System (HIS) and medical records, and blood samples were collected from all participants before treatment using epitope detection in monocytes (EDIM) technology 60 min after their last meal. Descriptive statistics and receiver operating characteristic (ROC) curves were used to compare the diagnostic performance of Apo10, TKTL1, and APT to those of conventional cervical cancer biomarkers (CEA, CA125, and SCC-A).

**Results:**

Most of the enrolled patients with cervical cancer had early-stage disease (70%) and squamous cell histology (84.9%). The Apo10, TKTL1, and APT levels were significantly higher in the cervical cancer group than in the control group (Apo10:139 vs. 132, TKTL1:121 vs. 114, APT: 260 vs. 246). We also found that Apo10, TKTL1, and APT showed superior diagnostic performance (AUC: 0.864, 0.865, 0.905) compared to traditional markers (CEA: 0.690, CA125: 0.594, SCC-A: 0.806). Sensitivity analysis revealed APT maintained high diagnostic value across tumor stages and in both HPV-negative (AUC = 0.967) and TCT-negative (AUC = 0.958) subgroups.

**Conclusion:**

Apo10, TKTL1, and APT outperform conventional biomarkers in detecting cervical cancer and may serve as reliable diagnostic indicators.

## Introduction

Cervical cancer ranks as the fourth leading cause of both incidence and mortality among women worldwide. In 2022, an estimated 660,000 new cases and 350,000 deaths occurred globally. It remains the most common type of cancer in 25 countries and the leading cause of cancer-related death among women in 37 countries. The incidence and mortality rates of cervical cancer show more than tenfold variation across different regions of the world [1]. Cervical cancer rates vary widely across regions, mainly due to disparities in screening availability and the prevalence of human papillomavirus (HPV) infection [2].

For many years, the Papanicolaou (Pap) test has been the gold standard for cervical cancer screening, demonstrating marked effectiveness and lowering the incidence of cervical cancer by up to 90% and mortality by 90% [3]. However, this cytological screening method is constrained by a sensitivity of only approximately 50%, with a notable proportion of specimens deemed inadequate [3]. The HPV test has been introduced as a screening tool, as HPV deoxyribonucleic acid (DNA) is present in almost all cervical cancers, and it has demonstrated higher sensitivity for high-grade cervical intraepithelial neoplasia (CIN2+) than that achieved by cytology in several studies [4]. Although HPV testing effectively identifies viral infection, its clinical utility may be limited by the inability to distinguish between transient infections and those likely to progress to precancerous lesions, potentially resulting in unnecessary patient anxiety and clinical interventions [5].

The use of flow cytometry to detect Apo10 and TKTL1 in blood macrophages (epitope detection in macrophages (EDIM)) is a new method for the diagnosis of noninvasive cancer [6]. Macrophages are a type of antigen-presenting cell (APC) that play important roles in the innate immune system. Macrophages engulf tumor cells, and their fragments at the beginning of the lesions become cancerous. After engulfing the tumor cells, the macrophages leave the tumor and move into the blood through the vascular system [7, 8]. The tumor substances in the macrophages are not diluted by blood but become highly concentrated in the macrophages due to their APC characteristics, which is also one of the key reasons for the high sensitivity and specificity of EDIM in detecting early-stage cancer [9, 10].

As mentioned previously, Apo10 and TKTL1 are two biomarkers that are commonly used in EDIM detection systems. Apo10 is an antigenic epitope of DNaseX (deoxyribonuclease) that plays a key role in cell apoptosis [8]. Compared with normal cells, cancer cells proliferate rapidly because they can trigger or possess defective programmed cell death (apoptosis) mechanisms. Thus, the accumulation of Apo10 in cells indicates blocked apoptosis, which can be used as a marker for detecting the development of tumors/proliferative diseases. In contrast, TKTL1 is a key enzyme of the non-oxidative branch of the pentose phosphate pathway (PPP) and is a structurally distinct transketolase-like enzyme lacking typical regulatory regions, which enhances its role in tumor metabolic reprogramming and cancer progression. It catalyzes reactions essential for nucleotide biosynthesis, antioxidant defense, and metabolic rewiring under hypoxia. Overexpression of TKTL1 leads to increased glycolysis (the Warburg effect), supporting rapid cancer cell proliferation, acidification of the tumor microenvironment, immune evasion, and resistance to apoptosis [11]. In cervical cancer, TKTL1 overexpression correlates with progression from low-grade lesions to high-grade neoplasia and invasive cancer [12, 13]. This metabolic shift is driven via pathways like AKT/PFKFB3, promoting proliferation, migration, and invasiveness in cervical cancer cells. TKTL1 has also been shown to distinguish between transient and persistent high risk HPV infections, thereby identifying women at increased risk of cervical neoplasia [14]. Therefore, TKTL1 is not only a biomarker of cancer presence but reflects a core driver of cervical carcinogenesis, making it highly relevant diagnostically and potentially therapeutically to cervical especially compared to HPV testing [15].

Apo10 tends to be detectable in the early stages of tumor development, whereas TKTL1 expression typically increases in parallel with tumor progression and invasiveness. Hence, evaluating both markers in tandem provides valuable insight into the malignant potential of tumors. Prior studies have shown that the combined measurement of serum Apo10 and TKTL1 levels can effectively differentiate patients with oral squamous cell carcinoma, those with prostate cancer, and healthy individuals, achieving a sensitivity of 95.8% and a specificity of 97.3% [9, 16], highlighting their potential utility as early tumor biomarkers. Our earlier investigations also confirmed that the EDIM test can accurately distinguish early-stage lung cancer from benign pulmonary nodules and healthy controls, with similar effectiveness in the detection of early breast cancer compared with benign lesions and noncancerous individuals [17, 18]. Nonetheless, the diagnostic utility of Apo10 and TKTL1 in the early detection of cervical cancer warrants further investigation.

## Methods

### Participants and ethics statement

From November 27, 2020, to August 23, 2023, patients aged 18–70 years with histologically confirmed cervical cancer were enrolled from the Department of Gynecologic Oncology at Sun Yat-sen University Cancer Center. An age-matched control group that was diagnosed with a normal or inflammatory cervix by HPV screening and the ThinPrep Cytologic Test (TCT) at the Cancer Prevention Center of SYSUCC was also enrolled. The histopathological findings were verified by two professional pathologists with associate senior titles and above. The study protocol was approved by the Institutional Review Board of Sun Yat-sen University Cancer Center (SYSUCC, ID: G2022-005-01). All patients provided written informed consent before enrollment.

The inclusion criteria for all participants were as follows:

- No pregnancy and no history of vaginal medication or irrigation within 7 days prior to enrollment;
- Patients who provided written informed consent after agreeing to participate, along with their family members.

The exclusion criteria were as follows:

- History of pelvic chemoradiotherapy;
- History of cervical or uterine surgery;
- Use of antibiotics within the past 7 days;
- Menstruation at the time of enrollment.

### APO10 and TKTL1 testing

We collected 2.7 mL of EDTA-anticoagulation venous blood from all the participants 60 min after their last meal and before their treatment for the cervical cancer group and before their physical examinations for the control group. The blood samples were stored at room temperature (15–25 °C) before testing. IntraPrep Permeabilization Reagent (Beckman Coulter, Krefeld, Germany) was used for intracellular staining, which was performed with antibodies against CD14 (OFC-14D) and CD16 (Hi-16a). After permeabilization, two intracellular antibodies, TKTL1 (phycoerythrin [PE]-conjugated, provided by Zyagnum AG, Pfungstadt, Germany) and Apo10 (fluorescein isothiocyanate [FITC], provided by Zyagnum AG, Pfungstadt, Germany), were added. One thousand macrophages (CD14+/CD16+) were selected, and analyses were performed using BD FACSDiva software v8.0 (BD Biosciences, Heidelberg, Germany). All the incubations in this protocol were conducted at room temperature in the dark. The EDIM scores of the participants were obtained by multiplying the proportion of CD14+/CD16 + cells containing Apo10 and TKTL1 by ten. The EDIM-combined scores were derived by adding the two scores for Apo10 and TKTL1 (APT). The intra-assay variability was assessed by calculating the coefficient of variation (CV) for repeated measurements of Apo10, TKTL1, and APT within the same assay batch. Due to the time-sensitive nature of the samples (all samples were processed within 30 h after collection to ensure biomarker stability), inter-assay (between-batch) variability was not evaluated as all samples were analyzed in a single continuous process to maintain consistency.

### Measurement of other cervical cancer-related biomarkers

Like Apo10 and TKTL1, serum tumor markers were prospectively measured before the start of therapy for the patients with cervical cancer and before their physical examinations for healthy controls. Carcinoembryonic antigen (CEA) was detected using a chemiluminescence method (Cobas e801, Roche Diagnostics, Germany). Carbohydrate antigen 125 (CA125), carbohydrate antigen 19 − 9 (CA199), and squamous cell carcinoma antigen (SCC-A) were detected using an electrochemical luminescence method (Cobas e801, Roche Diagnostics, Germany) with commercial assay kits according to the manufacturer’s instructions. The reference intervals of CEA, CA125, CA199, and SCC-A in the serum were 0–5.0 ng/mL, 0–35 U/mL, 0–35 U/mL, and 0–2.7 ng/mL, respectively.

### HPV and TCT results for the cervical cancer and control groups

The healthy control group underwent an HPV genotyping test at our institution, which included 23 human papillomavirus subtypes, including 17 high-risk and 6 low-risk genotypes. We defined HPV negativity as negative for all 23 HPV subtypes and HPV positivity as positive for any one HPV subtype. In addition, some of the participants in the healthy group underwent TCT examination, and most of the patients in the cervical cancer group had undergone TCT examination at a local hospital before admission. According to the third edition of the Bethesda system (TBS) for squamous epithelial lesions, we defined patients as TCT negative when the result was negative for intraepithelial lesions or malignancy (NILM), inflammation or no abnormalities. Other outcomes included atypical squamous cells (ASCs), atypical squamous cells of undetermined significance (ASCs-US), atypical squamous cells cannot exclude high-grade lesions (ASCs-H), low-grade squamous intraepithelial lesions (LSILs), high-grade squamous intraepithelial lesions (HSILs), and squamous cell carcinomas (SCCs), which were judged as TCT positive.

### Clinicopathological characteristics of patients with cervical cancer

This study collected clinical and pathological data in a standardized manner from the electronic medical records system at our institution. Baseline characteristics included demographic parameters such as age at diagnosis. Tumor pathological features, including histological type and differentiation grade, were documented according to the latest World Health Organization (WHO) diagnostic criteria. Clinical stage was determined based on the revised 2018 International Federation of Gynecology and Obstetrics (FIGO) staging guidelines. We also collected key clinical indicators, including HPV infection status and TCT results. All pathological diagnoses were independently verified by board-certified pathologists at our institution to ensure data accuracy and reliability.

### Statistical analysis

All analyses were performed via R Studio (Version 2023.06.0 + 421, Copyright © 2022 by Posit Software, PBC) software. The means and standard deviations (± SDs) or medians and interquartile ranges are used to describe the distributions of Apo10, TKTL1, APT and other cervical cancer-related biomarker levels. A series of independent-sample t tests were used to compare the means of Apo10, TKTL1, APT, and other cancer biomarkers between the cancer and noncancer groups. Then, ROC curves were generated, and the area under the curve (AUROC) was calculated to compare the diagnostic values of Apo10, TKTL1, APT and traditional cancer biomarkers in distinguishing patients with cervical cancer from controls. Logistic regression analysis was performed to determine the optimal cutoff values of Apo10, TKTL1, APT and the other biomarkers for screening early-stage cervical cancer. Several subgroup analyses were performed to test the robustness of Apo10, TKTL1, and APT in screening cervical cancers. Additionally, post-hoc statistical power calculations for ROC analyses were conducted in predefined subgroups, based on the observed AUC values and case/control sample sizes. Differences were considered statistically significant at *P* < 0.05.

## Results

### Clinical characteristics of the study participants

Table 1 shows the comparisons between the patients with cervical cancer and the healthy controls. A total of 152 patients with cervical cancer and 152 healthy controls were included in this study. The mean ages of the healthy controls and patients with cervical cancer were 49.5 ± 9.90 years and 50.6 ± 10.0 years, respectively, with no statistically significant difference. As detailed in Table 1, compared with healthy controls, patients with cervical cancer presented significantly elevated levels of Apo10 (139 ± 5.03 vs. 132 ± 4.24), TKTL1 (121 ± 5.72 vs. 114 ± 4.11), and APT (260 ± 8.69 vs. 246 ± 7.03) (all *P* < 0.001). Moreover, the levels of CEA, SCC-A, and CA125 were significantly greater in the patients with cervical cancer than in the healthy controls (all *P* < 0.05), whereas CA199 was not significantly different between the groups (*P* = 0.220). Among the patients with cervical cancer, 88 (57.9%) were diagnosed with stage I cervical cancer, and 29 (19.1%) were diagnosed with stage II cervical cancer, with the vast majority being cervical squamous cell carcinomas. Compared with the control group, patients with cervical cancer demonstrated significantly higher rates of both HPV positivity (59.2% vs. 11.2%) and TCT positivity (41.4% vs. 3.9%).

**Table 1:** Comparison of baseline characteristics between patients with cervical cancer and controls

Variables	Healthy Controls (*N* = 152)	Cervical Cancer (*N* = 152)	*P* value
Age			
Mean (SD)	49.5 (9.90)	50.6 (10.0)	0.320
Median [Min, Max]	51.0 [26.0, 72.0]	52.0 [24.0, 71.0]	
Apo10			
Mean (SD)	132 (4.24)	139 (5.03)	< 0.001
Median [Min, Max]	133 [120, 142]	139 [121, 154]	
TKTL1			
Mean (SD)	114 (4.11)	121 (5.72)	< 0.001
Median [Min, Max]	114 [104, 124]	120 [110, 147]	
APT			
Mean (SD)	246 (7.03)	260 (8.69)	< 0.001
Median [Min, Max]	247 [227, 261]	258 [231, 294]	
CEA			
Mean (SD)	1.51 (1.03)	3.20 (4.33)	< 0.001
Median [Min, Max]	1.22 [0.300, 7.08]	1.87 [0.193, 41.7]	
CA199			
Mean (SD)	12.4 (10.4)	15.5 (28.0)	0.220
Median [Min, Max]	9.42 [2.00, 86.5]	10.5 [1.28, 319]	
Missing	0 (0%)	16 (10.5%)	
SCC-A			
Mean (SD)	1.10 (0.525)	5.63 (8.07)	< 0.001
Median [Min, Max]	0.950 [0.360, 3.40]	2.12 [0.450, 54.5]	
Missing	0 (0%)	2 (1.3%)	
CA125			
Mean (SD)	14.3 (11.3)	17.8 (16.5)	0.036
Median [Min, Max]	11.5 [3.70, 86.8]	14.4 [3.85, 125]	
HPV (%)			
HPV Positive	17 (11.2)	90 (59.2)	< 0.001
HPV Negative	100 (65.8)	7 (4.6)	
Missing	35 (23.0)	55 (36.2)	
Stage (%)			
0	152(100)	0(0)	< 0.001
I	0(0)	88(57.9)	
II	0(0)	29(19.1)	
III	0(0)	34(22.4)	
IV	0(0)	1(0.7)	
TCT (%)			
TCT Negative	124(81.6)	6(3.9)	< 0.001
TCT Positive	2(1.3)	63(41.4)	
Missing	26(17.1)	83(54.6)	
Pathological differentiation (%)			
Grade 1	0(0)	61(40.1)	< 0.001
Grade 2	0(0)	56(36.8)	
Grade 3	0(0)	35(23.0)	
Healthy Control	152(100)	0(0)	
Pathological type (%)			
S	0(0)	129 (84.9)	< 0.001
A&O	0(0)	23 (15.1)	
Healthy Control	152(100)	0(0)	

Note: S = squamous cell carcinomas; A&O = adenocarcinoma/others

### Comparison of Apo10, TKTL1, and APT levels in the cervical cancer and healthy control groups

**Fig. 1:**
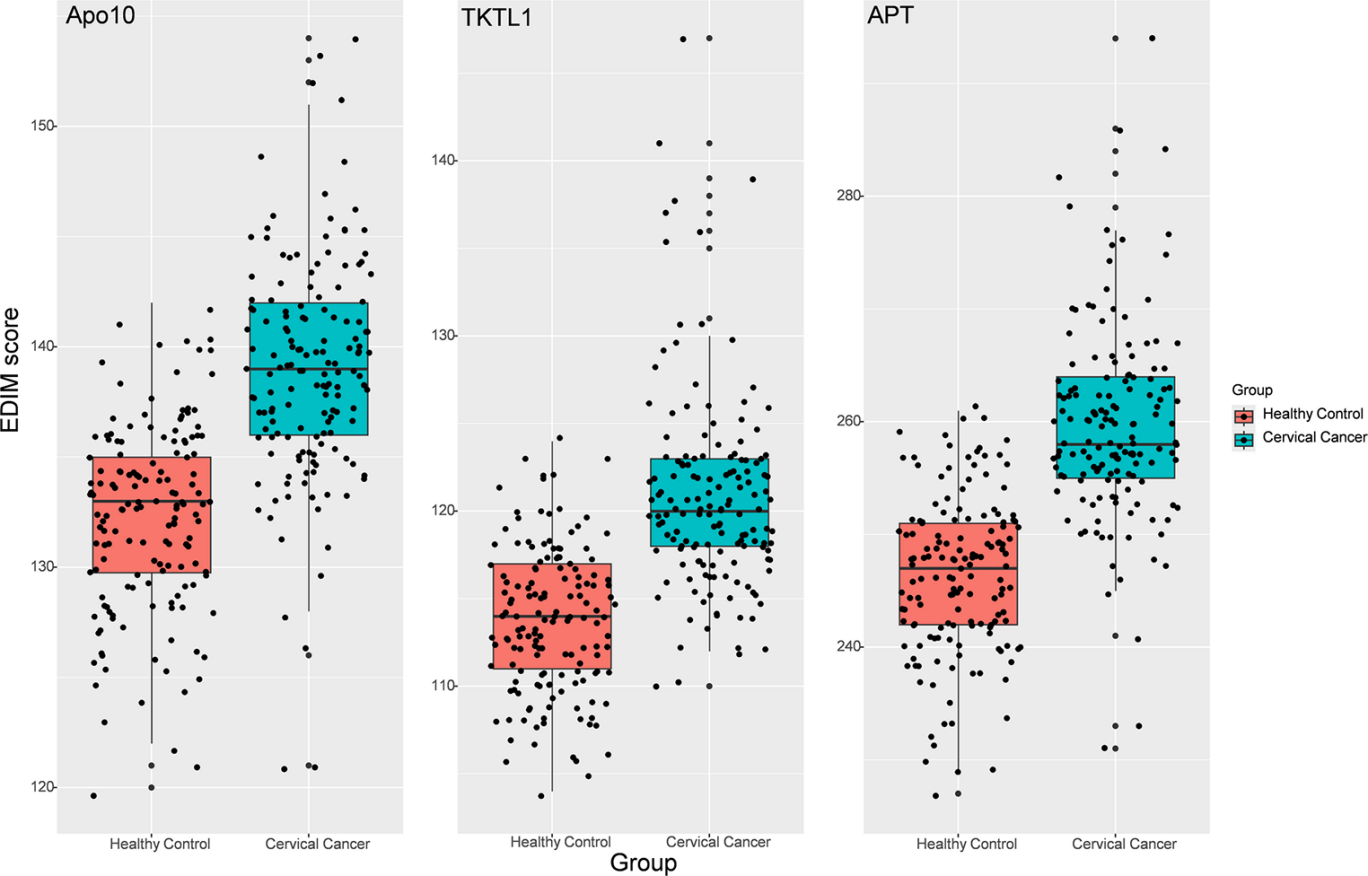
Distributions of Apo10, TKTL1, and APT in the cervical cancer and healthy control groups

Box plots (Fig. 1) were constructed to display the distributions of Apo10, TKTL1, and APT levels in the patients with cervical cancer and healthy controls. The median values of Apo10 (139 vs. 133), TKTL1 (120 vs. 114), and APT (258 vs. 247) were greater in the cervical cancer group than in the control group. The data points show a consistent upward shift across all three biomarkers in the cancer group. Intra-assay variability for Apo10, TKTL1, and APT was assessed, with mean coefficients of variation (CV) of 0.74%, 0.78%, and 0.54%, respectively (*n* = 1).

### Diagnostic value of Apo10, TKTL1, APT and other conventional biomarkers

**Fig. 2:**
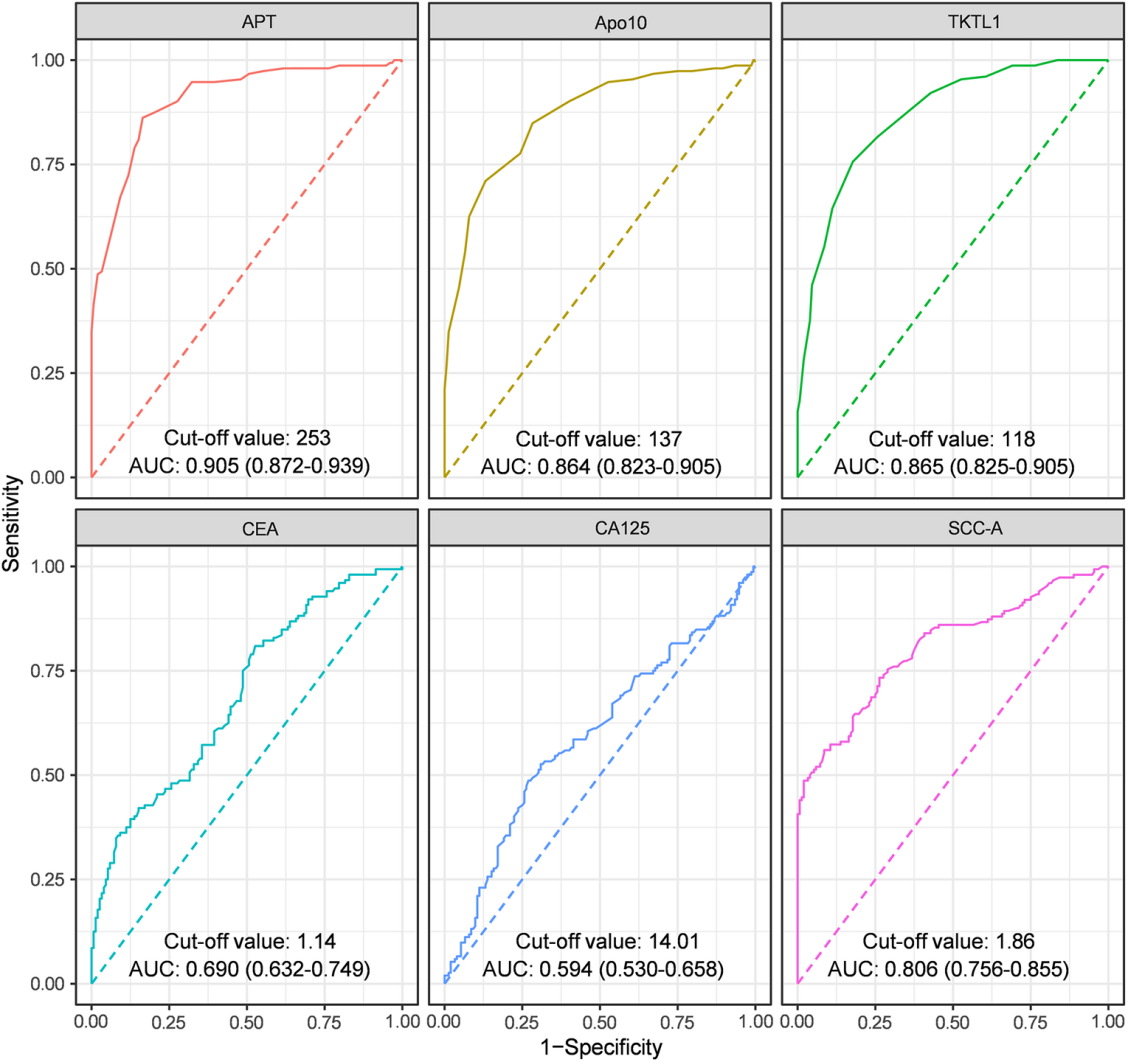
Performance of Apo10, TKTL1, APT, and other tumor biomarkers in differentiating patients with cervical cancer from controls

ROC curve analysis demonstrated that Apo10, TKTL1, and APT had superior diagnostic performance to conventional cervical cancer biomarkers (Fig. 2). The AUCs of Apo10, TKTL1, and APT in screening for cervical cancer were 0.864 (95% CI: 0.823–0.905), 0.865 (95% CI: 0.825–0.905), and 0.905 (95% CI: 0.872–0.939), respectively. The optimal cutoff values of Apo10, TKTL1, and APT were 137, 118, and 253, respectively. For the conventional biomarkers CEA, CA125, and SCC-A, SCC-A achieved the highest diagnostic value (AUC = 0.806, 95% CI: 0.756–0.855), followed by CEA (AUC = 0.690, 95% CI: 0.632–0.749) and CA125 (AUC = 0.594, 95% CI: 0.530–0.658). The optimal cutoff values of CEA, CA125, and SCC-A were 1.14, 14.01, and 1.86, respectively. As shown in Table 2, the sensitivity, specificity, positive predictive value (PPV), and negative predictive value (NPV) of Apo10, TKTL1, and APT were summarized, with APT demonstrating a sensitivity of 86.2%, specificity of 83.6%, PPV of 84.0%, and NPV of 85.8%.

**Table 2:** Diagnostic performance of Apo10, TKTL1, and APT

Variable	AUC (95% CI)	Accuracy	Sensitivity	Specificity	PPV	NPV	Cutoff
Apo10	0.864 (0.823–0.905)	0.789	0.711	0.868	0.844	0.750	137
TKTL1	0.865 (0.825–0.905)	0.789	0.757	0.822	0.810	0.772	118
APT	0.905 (0.872–0.939)	0.849	0.862	0.836	0.840	0.858	253

### Sensitivity analysis

**Fig. 3:**
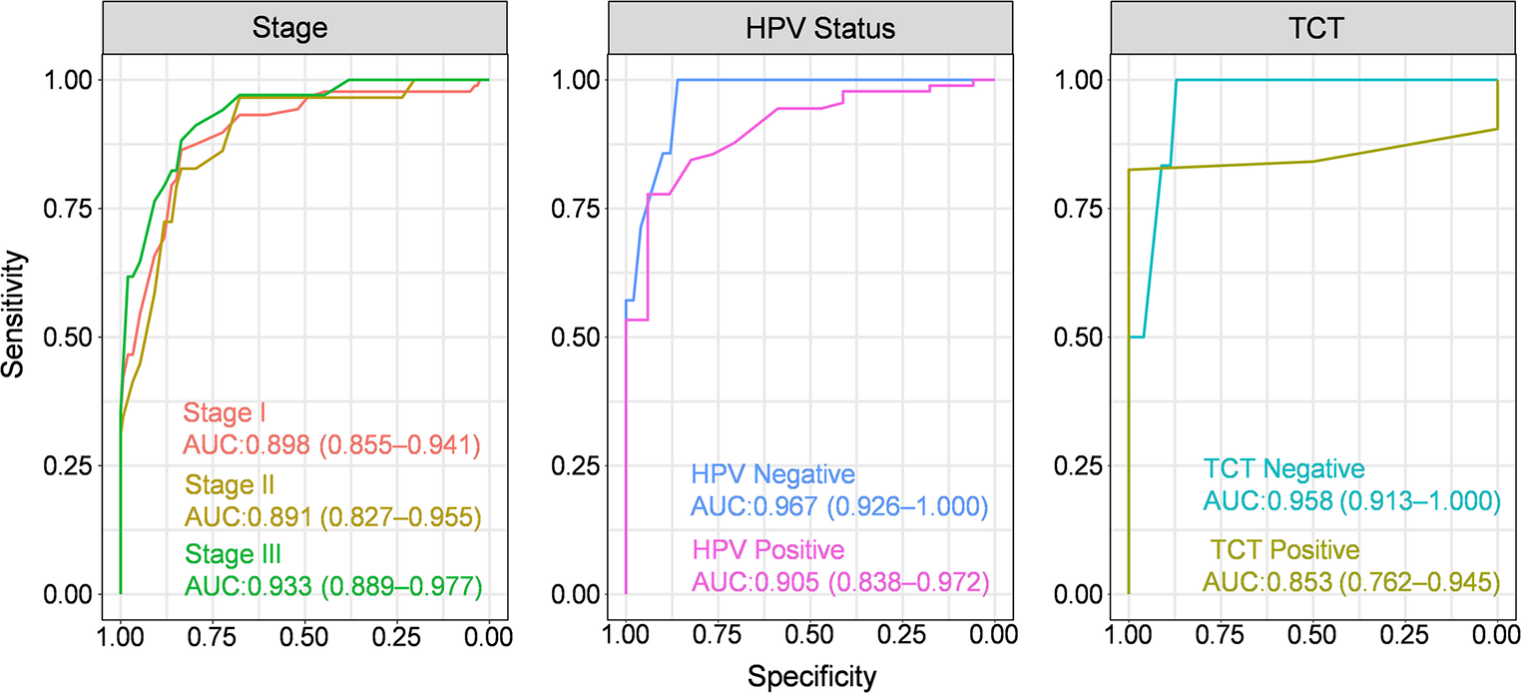
Comparative ROC analysis by tumor stage, HPV status, and TCT results

We conducted a sensitivity analysis of the diagnostic performance of APT for cervical cancer across different disease stages and HPV and TCT statuses (Fig. 3). In the stage-stratified analysis, APT demonstrated robust performance, with AUC values of 0.898 (95% CI: 0.855–0.941) for stage I, 0.891 (95% CI: 0.827–0.955) for stage II, and 0.933 (95% CI: 0.889–0.977) for stage III. Notably, all stages showed consistent sensitivity (0.836), whereas specificity progressively increased from 0.828 (stage II) to 0.864 (stage I) and 0.882 (stage III), with a uniform optimal cutoff value of 252.5 (Table 3). When stratified by HPV status, APT exhibited excellent diagnostic accuracy for both HPV-negative (AUC = 0.967, 95% CI: 0.926–1.000) and HPV-positive (AUC = 0.905, 95% CI: 0.838–0.972) patients. The test showed greater specificity in HPV-negative patients (1.000 vs. 0.778) but greater sensitivity in HPV-positive patients (0.941 vs. 0.860), with an optimal cutoff of 254.5 for both groups (Table 3). Analysis by TCT status revealed AUC values of 0.958 (95% CI: 0.913–1.000) for TCT-negative patients and 0.853 (95% CI: 0.762–0.945) for TCT-positive patients. APT demonstrated perfect sensitivity (1.000) in TCT-negative cases and perfect specificity (1.000) in TCT-positive cases, with optimal cutoff values of 254.5 and 252.0, respectively (Table 3).

**Table 3:** Diagnostic performance of APT across tumor stages (I–III), HPV infection status, and TCT screening results

Variable	AUC (95% CI)	Accuracy	Sensitivity	Specificity	PPV	NPV	Cutoff
Stage I	0.898 (0.855–0.941)	0.846	0.836	0.864	0.914	0.752	252.5
Stage II	0.891 (0.827–0.955)	0.834	0.836	0.828	0.962	0.490	252.5
Stage III	0.933 (0.889–0.977)	0.844	0.836	0.882	0.969	0.545	252.5
HPV Negative	0.967 (0.926–1.000)	0.869	0.860	1.000	1.000	0.333	254.5
HPV Positive	0.905 (0.838–0.972)	0.804	0.941	0.778	0.444	0.986	254.5
TCT Negative	0.958 (0.913–1)	0.877	1	0.871	0.273	1	254.5
TCT Positive	0.853 (0.762–0.945)	0.831	0.825	1	1	0.154	252

Note: PPV = positive predictive value; NPV = negative predictive value

**Fig. 4:**
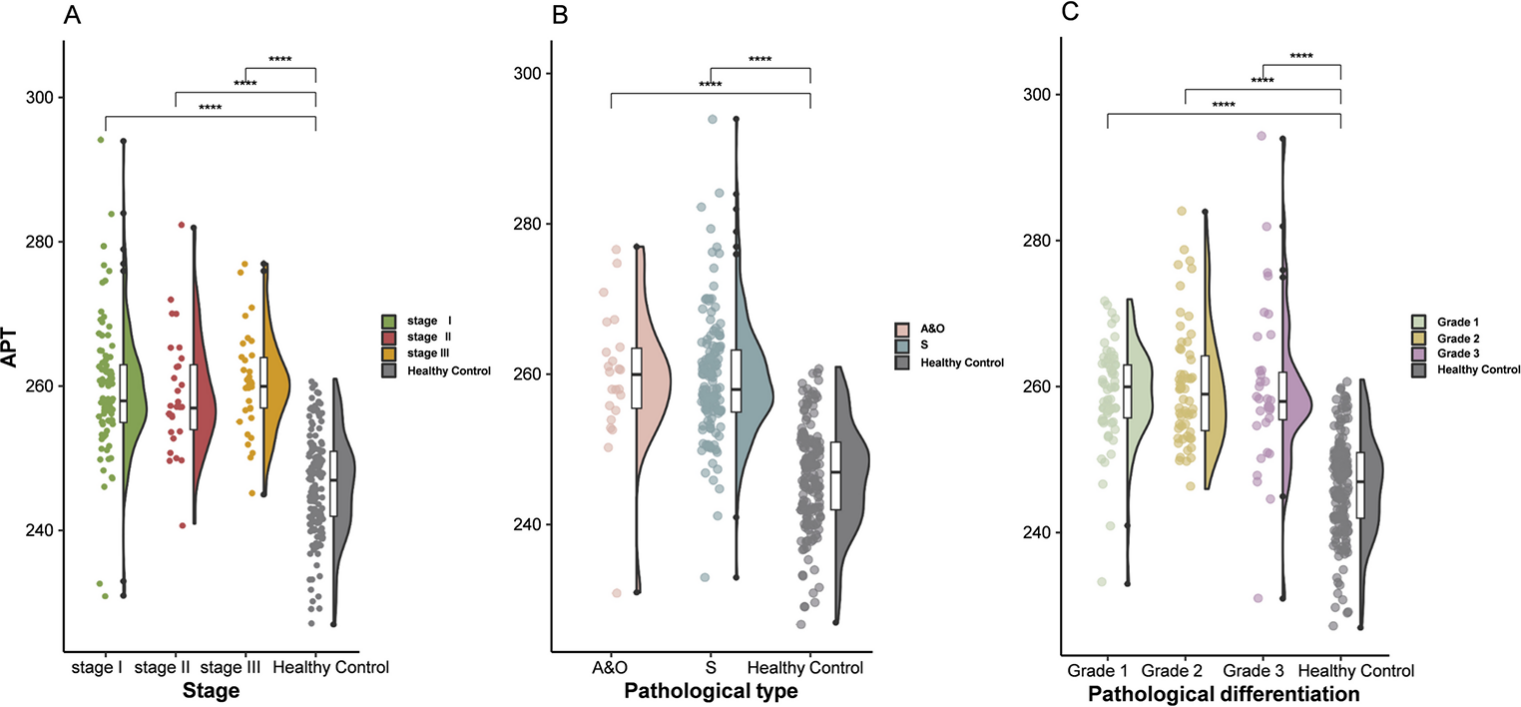
Distribution patterns of APT biomarkers across cervical cancer stages, pathological types, and differentiation grades compared with those in healthy controls

The analysis revealed consistently elevated APT levels in patients with cervical cancer across specific clinicopathological characteristics compared with those in healthy controls (Fig. 4). The stage-stratified analysis revealed that patients with all cancer stages had higher APT levels than the controls did, with stage III patients having the highest median value among the cancer groups. In the pathological type analysis, both squamous cell carcinomas (S) and adenocarcinoma/other carcinomas (A&O) displayed significantly elevated APT expression compared with that in healthy individuals. Most notably, the differentiation analysis revealed that well-differentiated tumors (Grade 1) presented the highest median APT values among all differentiation grades, followed by moderately differentiated (Grade 2) and poorly differentiated tumors (Grade 3), with all three grades maintaining values substantially above those of healthy controls.

## Discussion

In this study, we focused on developing a diagnostic approach based on blood macrophage biomarkers, including Apo10, TKTL1, and APT, to increase the accuracy of cervical cancer detection and reliably discriminate patients with malignancies from healthy individuals. Compared with conventional biomarkers such as CEA, CA125 and SCC-A, APT has greater diagnostic value and stronger efficacy in differentiating patients with cervical cancer from healthy people. Notably, these findings remained consistent and robust regardless of clinicopathological variations, spanning stage, HPV/TCT status, pathological subtype and tumor differentiation grade.

The diagnostic superiority of Apo10, TKTL1, and particularly APT was underscored by APT’s AUROC (0.905), sensitivity (86.2%), and specificity (83.6%), which significantly exceeded those of conventional markers such as CEA, CA125, and SCC-A. Although a small proportion of the cases or controls would be mis-classified, the observed false-negative rate and false-positive rate of our approach fall within clinically acceptable ranges, given that most cervical cancer cases in our study were early-stage. Further research should focus on optimizing APT’s performance by addressing technical challenges such as reducing inflammation-related interference and enhancing extracellular staining techniques to minimize misclassification rates. Nevertheless, our results highlight the enhanced accuracy of macrophage-derived biomarkers and support their potential utility in differentiating cervical cancer from noncancerous conditions, thereby contributing to earlier and more reliable diagnoses. Subgroup analysis further validated the robustness and generalizability of APT in screening cervical cancer across a variety of clinical aspects such as HPV and TCT statuses, clinical stages, pathological types, and degrees of differentiation.

Cervical cancer is a gynecologic malignancy strongly associated with high-risk HPV infection, and HPV-negative cervical cancer accounts for approximately 3–8% of all cases [19]. Although the vast majority of cervical squamous cell carcinomas are HPV positive, the proportion of HPV-negative cases is significantly greater in cervical adenocarcinomas, ranging from 15 to 38% [20]. Unlike HPV-dependent carcinogenesis, HPV-negative tumors often rely on alternative oncogenic pathways, such as aberrant glucose metabolism and hypoxia-induced signaling. These pathways may drive the aggressive clinical behavior of HPV-negative cervical cancers, which present at more advanced FIGO stages, exhibit greater molecular heterogeneity, and are associated with poorer clinical outcomes, making early detection even more crucial [21]. In this study, we found APT especially TKTL1 could not only effectively screen HPV-positive cervical cancer patients, but also HPV-negative patients, thus filling a diagnostic gap left by HPV testing. This can be attributed to that TKTL1 capture tumors driven by virus-independent mechanisms (e.g., metabolic reprogramming). Although the sample size of HPV-negative participants was relatively small, our post-hoc power analysis confirmed adequate statistical power (power = 1) for this subgroup, supporting the reliability of the observed results.

Unlike TKTL1, Apo10 is a specific protein isoform, which has been shown to accumulate abnormally when apoptosis is impaired, especially due to suppressed DNA fragmentation. This allows damaged but nonapoptotic cells to escape immune surveillance, accumulate mutations, and gain survival advantages—events that may precede morphological transformation [6]. The combined expression of Apo10 and TKTL1 represent a sequential oncogenic cascade in HPV-negative cervical cancer, linking impaired apoptosis to metabolic rewiring and this mechanism may represent an intrinsic pathway that functionally parallels virus-driven oncogenesis. APT testing could capture tumor-associated antigens via immunological mechanisms, offering a real-time, functional snapshot of malignancy, making it particularly useful for detecting cervical cancers or precancerous lesions that are not identified by standard virological screening method.

As mentioned before, conventional cytological methods such as TCT, face several limitations, including sampling errors due to anatomical complexity or uneven lesion distribution and diagnostic variability stemming from differences in operator technique and pathologist interpretation. The EDIM-based Apo10/TKTL1 assay relies on standardized flow cytometry protocols and requires only 2.7 mL of venous blood, which remains stable at room temperature (15–25 °C). These features simplify sample collection, transport, and analysis, making the assay especially suitable for deployment in primary care settings or regions with limited medical resources. By minimizing subjectivity and enhancing diagnostic coverage, this approach offers a practical and equitable solution to current screening gaps.

Nonetheless, this study has certain limitations. The study was conducted at a single institution, which may limit the generalizability of the results. Additionally, although the control group was age-matched, potential confounding factors such as hormonal status, smoking history, and immune-related conditions were not fully controlled and may have influenced biomarker expression. Despite these constraints, current evidence suggests that the EDIM blood test holds promise as a noninvasive adjunct to conventional screening methods. In resource-limited settings, early detection could be enhanced by overcoming the limitations of cytology and virology-based approaches. In more advanced health care systems, it may provide diagnostic clarity in HPV-negative or cytologically indeterminate cases. However, these potential applications remain exploratory and must be rigorously validated before clinical implementation.

## Data Availability

The authenticity of this article has been validated by uploading the key raw data onto the Research Data Deposit public platform (www.researchdata.org.cn), with the approval RDD number as RDDA2025190850.
